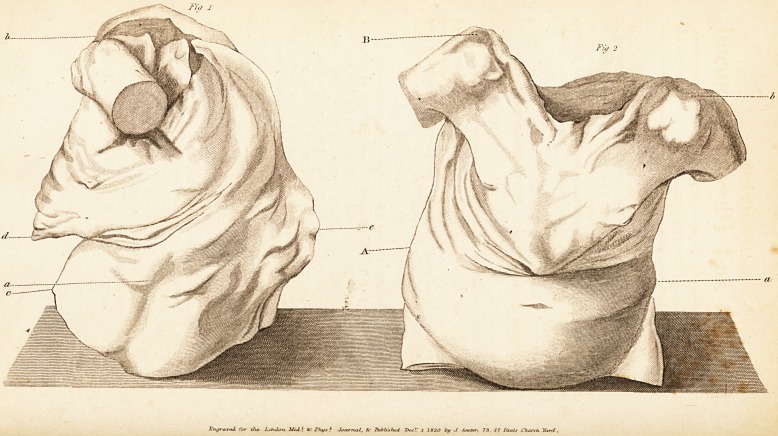# Remarks upon the Different Appearances of the Back, Breast, and Ribs, in Persons Affected with Spinal Diseases; and on the Injurious Effects of Spinal Distortion on the Sanguineous Circulation and the Internal Viscera

**Published:** 1820-12

**Authors:** Edward Harrison


THE LONDON
### Medical and Physical Journal.

### Original Communications, Select Observations, etc.

FOR THE LONDON MEDICAL AND PHYSICAL JOURNAL.

### *Remarks upon the different Appearances of the Back, Breast, and Ribs, in Persons affected with Spinal Diseases; and on the injurious Effects of Spinal Distortion on the Sanguineous Circulation and the Internal Viscera.* By EDWARD HARRISON, M.D. F.R.A.S. ED. &C.

[In continuation from page 378.]

*First Case of Distorted Spine.*

MARY ANN RAFTER, aged fifteen the 3d of January last, weighs only fifty-four pounds, and is four feet one inch in height. She always leans towards the left side, which she supports by a strong crutch placed under the arm-pit. She met with an accident, when only eight years old, by tumbling upon the pavement out of a window in the first floor: she was much bruised, and in consequence fell into bad health. Three months afterwards she was first discovered to be crooked in the back and breast. For the removal of this deformity, she became a patient in the Brunswick-street Hospital in Dublin, where she underwent the usual treatment, by issues and caustics applied to the upper part of her back, during the period of seven months. She left the hospital unrelieved, and has since that time never applied for further medical assistance. Pulse 80, small and feeble. Is always costive. She sleeps well; has a good appetite; and can bear a great deal of fatigue, though her breathing is easily disturbed and becomes very distressing on using slight exertions. Height of her side from the spine of the ileum(*a*),* to the top of the shoulder (*b*), on the left side, six inches and a quarter; ditto (A), from ditto (B), on the right

* See the engraved Prints.

side, seven inches and a half. Distance from the umbilicus (*c*), to the point of the sternum (*d*), two inches. Circumference of the body at the point of the sternum (*e*), round the most prominent vertebræ, twenty-nine inches.—*July* 14*th*, 1820.

On examining her this forenoon, the spine was found to be straighter, sensibly elongated, and its several parts had become more distinct. The five lumbar vertebræ were seen raised into an elevated arch. Above them, the bones were depressed. The shoulders, and spine lying between them, were pushed outwards, making a strongly-marked hump. The lower cervical bones are too protuberant. The sternum is little altered. She breathes with difficulty in the horizontal posture, and the abdominal muscles act powerfully to assist the respiration. The belly is less tumid; and the spines of the ossa ilia, relieved from the load of flesh which hung over them, are clearly visible. Height on the left side, as above, nine inches and a quarter; ditto on the right side, eleven and a quarter; length of spine, from the nape to the clift, fifteen inches and a half; circumference round the chest, as above, twenty-seven and three-quarters.—*July* 16*th*.

The respiration is quite easy, and she is perfectly well in health. Height on the left side, ten inches; ditto on the right side, eleven inches and a half. The circumference from the point of the sternum now falls into the hollow of the back, and measures twenty-six inches. The improved shape of the spine is visible in every part. The cervical bones were at first thrust confusedly together, leaving very little space between the top of the chest and head. They are already restored in great measure to their natural beds, by which the neck is considerably elongated. The hump includes two or three of the cervical, with five or six of the upper dorsal, vertebræ, and also comprehends the back parts of both scapulæ, making altogether a very unsightly protuberance. The lumbar vertebræ are all engaged in forming a considerable posterior, and somewhat lateral, curve, in which the bodies on the left side are thrust downwards. On the right, they are forced obliquely outwards; at the same time, they lean towards the top of the sacrum in such a way that the transverse processes of the two last vertebræ are forced partly over the posterior spinous process of the right os ilium. It is chiefly owing to the particular shape of the arch, and its inclination to the right loin, that this side is longer than the left. The lower dorsal bones, pressed together, were at first almost concealed between the hump and the lumbar arch.—*July* 31*st*.

She is in the best possible health, and gains flesh daily. The height on the left side, as formerly taken, is eleven inches and a half; ditto on the right side, as ditto, is twelve inches and a quarter; circumference, taken from the sternum, now surrounds the lower part of the hump, and is twenty-seven inches; distance from the umbilicus to the point of the sternum, five and a half inches.—*August 25th.*

She is in all respects well, and quite free from pain. She has gained a great deal of flesh and colour. The mammæ, which were flat and indistinct, have become full and prominent since she entered upon her present plan. The neck, which was at first concealed between her shoulders, is now raised above them, and is well formed. It is owing to this improved disposition in the shoulder points, that the length of the two sides approximate nearer to each other, and are a little reduced in length. Height of left side, ten inches and three-quarters; ditto of right side, eleven and a quarter inches; circumference, twenty-seven inches; distance of the umbilicus from the sternum, five inches; length of the spine, as formerly measured, sixteen inches; length of the whole person, four feet seven inches.—*Oct.* 31.

[*To be continued.*]

*Second Case.*

Mrs. A. B. aged twenty-six, of a sanguine temperament and fair complexion, complains of great and long-continued weakness and uneasiness in her back. Upon examination, I found the third, fourth, fifth, and sixth of the dorsal vertebræ much displaced, and projecting backwards in form of an arch. On slightly touching the skin on the right side near to the projection, she suffers a painful sensation, which always goes off when greater pressure is used. Upon coughing or sneezing, she constantly experiences a distressing tightness across the chest. Going up and down stairs always, and walking generally, produce great difficulty in breathing. She is liable to faint from slight exertions. Occasionally, some pricking and numbness are felt in the right foot for a few minutes. Little pain or tenderness is excited by pressing the spines or bodies of the vertebræ; nor does there appear to have ever been any inflammation in them. She first discovered a small projection in the back about six years since. As it was never accompanied with pain or tenderness, she continued to disregard it, under a supposition that nothing could be done for her relief; though she knew that the enlargement kept increasing, and that her height was visibly diminished. From this time exercise soon produced fatigue, and the trunk suffered most from it. She spent six weeks, in the spring of 1817, in a constant round of fashionable company; in which time the weakness of the back was so much increased, that a physician and surgeon of the greatest eminence in London were consulted. Four dorsal vertebræ were discovered to be much displaced, and the medical gentlemen said they could only be cured by anchylosis. A recumbent posture, according to Mr. Baynton's plan was advised; and the diseased arch was covered with emplast. galban. comp. Tonic and aperient medicines were also directed. This mode of treatment, though never strictly followed, has been persevered in without any benefit, for the last fourteen months. —*October 24th*, 1818.

The directions given on the 24th having been carefully followed, the two uppermost of the affected vertebræ have already sunk down so as to leave a great hollowness between them and the two lower ones. The distressing pain in the skin formerly mentioned is nearly gone; nor has she lately suffered much uneasiness of the right foot.—*Dec. 5th.*

The lowest vertebra in the curve recovered its natural situation six weeks since, and the other three are much fallen. The pain on the right side of the incurvation is quite gone. She no longer experiences any inconvenience from sneezing or coughing. The difficulty in breathing, and the disposition to faint, have entirely left her; as well as the tightness over the chest and stomach.—*March 21st*, 1819.

The two upper bones of the arch have obtained their proper places, and been stationary for the last two or three weeks.—*May 12th.*

All the bones are restored to their natural stations, leaving little prominence or other affection of the spine.—*July 25th.*

She has been lately permitted to rise from her mattress, and walk about the room for a quarter of an hour before and after dinner. She moves with ease, and finds no inconvenience from it. The health is extremely good.—*Sept. 20th.*

On returning home after an absence of four months, her friends were particularly struck with her improved appearance, and declared her to be considerably taller than at any former period. This opinion leads to a belief that the disorder begun earlier than the time above stated.—*Oct. 20th.*

The lady continues in very good health in every respect. The spine remains quite sound and well. Walking and carriage exercise afford her great pleasure, and are pursued without fatigue.—*Sept. 30th*, 1820.

### *Third Case.*

Mr. C. D. aged twenty-four, of a delicate habit, has been a long time indisposed. He is subject to severe palpitations of the heart from slight causes, great tightness over the stomach, and want of appetite. He swallows with inconvenience, and reading aloud soon tires him. The eye-sight has gradually got worse for several years past. He used to amuse himself with gardening, but has been obliged to desist, because, on stooping, his face became uncomfortably swollen and red for a long time afterwards. He also felt a dull heavy pain at the upper and internal part of his head. He suffers great uneasiness in the right side whenever even slight pressure is made on the edge of the ribs, or immediately below them. All the false ribs are flat and protuberant. The liver has, in consequence, been forced upwards, and driven out of its proper situation. I impute the pain, fulness, and prominence, to a derangement in the functions of the liver, from its having been displaced along with the ribs. He has taken an aversion to common diet, and relishes only fat meats, butter, or food prepared with warm condiments. Left arm very weak, and often nearly insensible. The lower limbs are numb and cold; they are also subject to clammy perspirations and convulsive twitchings. Bowels constipated. Urine very turbid, of an extraordinary colour and disagreeable smell. Pulse frequent. The whole of the spine lying between the shoulders is considerably arched, and rises so much above them as to be quite unsightly. The small of the back is disagreeably hollow. This prominence is occasioned by the sub-luxation of three of the cervical, and five of the upper dorsal, vertebræ; all of which are tender, stand irregularly, and at unequal distances from each other. He imputes his complaint to the fatigue of riding on horseback when in weak health. He fainted repeatedly from the exertion. He has consulted several of the most eminent of the faculty within the last few years: no two of them entertained the same opinion of his case; some declared him to be consumptive, and recommended a mild climate; others referred his disorder to the liver, and others to the stomach.—*March 3d,* 1820.

The eight vertebræ formerly described have been for some time restored to their natural situations. The curve is entirely removed, and the part sunk into a grove of nearly an inch in depth below the shoulders. The hollowness in the loins is not more than natural. The spine, in consequence of these alterations, is become straight and remarkably well-shaped. The ribs on the right side have been made to resemble those on the left. The palpitations have entirely left him, and the tightness over his stomach is removed. The weakness and numbness are no longer felt in his arms and legs. Has not lately had spasms nor clammy sweats, and the feet are much warmer. His looks have considerably improved, and the urine is natural. He has taken an aversion to fat in every way, preferring lean meats, with a proper mixture of vegetables. The diet is natural, and appetite good.—*May 5th.*

He is quite well in health, and feels his back getting strength daily. The liver having fallen into its natural situation, the tenderness formerly mentioned is nearly gone: it had constantly troubled him for the last ten years.—*July 20th.*

The eye-sight is entirely restored. Deglutition is easy, and reading aloud no longer fatigues him. He returned home this day in very good health and spirits.—*Sept. 18th*, 1820.

*Fourth Case.*

Miss E. F. aged twenty, five feet four inches in height, of a very delicate constitution and in puny health, complains of great pain, general tension, and fulness in the abdomen on the right side, which is almost constant. She always awakes suddenly in the morning with an irresistible propensity to make water: this is voided with difficulty, in small quantities, and through the day with nearly continual calls. The bowels are always constipated, and require strong medicines to open them. Has very little appetite, and no power of digesting animal food. There is continual sickness, with the frequent discharge of a clear tasteless fluid. Has distressing flushes after eating. Swallows with great difficulty; even common-sized pills require to be cut into three or four. Almost perpetual head-ach, with great internal weight and external pain. Objects appear to dance before her eyes. The sight is much weakened, and the eyes are dull and dry. Has great difficulty in walking; is soon tired. Has a dragging and uneasy feel about the right hip and rim of her body. She often stumbles, and cannot bear the motion of a carriage. The arms and legs, more especially on the right side, appear black, mottled, and as if the blood were stagnant in them. Serious apprehensions of palsy have occasioned frequent alarm to her anxious and attentive relations, from the occasional numbness and insensibility of the right hand and leg. The ankles are often swelled. She is seldom free from chilblains and bunions. Glands in the neck much enlarged, and almost constant sore-throat. Menses appeared at fifteen; she has never been regular, nor has there been any show since February 1818. Fluor albus almost constant. The spirits are always depressed, and she sleeps very ill. In April last, a very eminent physician was consulted, who, after a minute and protracted investigation, declared her to be in a consumption.

On examination, I found all the vertebræ of the neck, back, and loins, tender and too prominent, especially the seven cervical and four upper dorsal. The former, from their size and appearance, her aunt calls the pullet-eggs. The sacrum has several lumps upon it, on the right side, about the size of walnuts. The right arm has drawn three of the lower cervical, and as many of the upper dorsal, bones considerably towards the same side. There is an opposite extensive curve in the loins. By these distortions, the right arm and right hip are sunk much below their fellows. This appears in the points of the shoulders and soles of the feet. To this is owing the frequent stumbling, the dragging and apparently increased length of the right lower limb, and not to any complaint in it. The ribs on the right side are very prominent, and have drawn the liver along with them. The sternum is unnaturally protuberant and narrow.—11*th May*, 1820.

Sleeps well. Countenance and spirits are much improved. Appetite better; the food taken agrees well. Pain, tension, and fulness of her body gone. Voids her urine only thrice daily; this is unattended with pain, and is in much greater quantities at a time. Swallows without difficulty. Vertebræ are much straighter, and less prominent. The cervical bones are so much sunk, that her aunt jocosely calls them the wren-eggs. Arms, hips, and bottoms of the feet, nearly correspond. The front of the chest is become round, and almost alike on both sides. There is no cough, and respiration is easy. The lumps upon the os sacrum have nearly disappeared.—23*th May*.

Appetite much better. Since the chest has assumed its natural appearance, the distressing abdominal sensations have entirely left her. The complexion is much brighter, more animated, and juvenile. It is presumed that the pain and fulness felt in the abdomen arose from impeded circulation in the contained viscera; and that her present ease is occasioned by the blood now passing freely through the vena portarum and replaced liver. The curves and protuberances are considerably reduced.—*June* 18*th*.

The spinal column keeps improving. She is easy, and well in health. Her difficulty in swallowing did not arise from any defect in the œsophagus, but from an imperfect contraction of the muscles engaged in deglutition: these could not act with correctness and precision till the vertebræ from which they originate had got replaced.—*Sept.* 8*th*.

[*To be continued.*]

*Fifth Case.*

I was consulted, July 1st, 1819, for Miss G. H. aged five years and a quarter, for a distorted spine. I found a gradual posterior projection of all the dorsal vertebræ to the eleventh inclusive. This bone rises a full inch above the back. The descent from it downwards is sudden and considerable, giving to the small of the back an unusual and disagreeable hollowness. The ribs, at their attachment to the vertebræ, have a rounded form. This part of the back swells out, appearing something like the breast of a well-trussed fowl. There is no tenderness in the part, or discolouration of the skin. She is of a sanguine and scrofulous habit, was always delicate, and often indisposed,

In the spring of 1817, she first refused to walk, complaining of weakness in the back, and of being always tired. She was constantly falling, which led to a suspicion that the back or limbs were affected. Nothing unusual could be discovered by her medical attendants, and she was sent to Margate for the improvement of her general health. In the following spring, she was observed frequently to lean towards the right side, and drag the right leg in walking. Alarmed at these appearances, recourse was had to a very eminent surgeon and the family apothecary. The vertebral column was, after careful examination, pronounced to be sound, and the symptoms were imputed to constitutional debility. Early in May, she removed into Berkshire for change of air; and, while there, amused herself with pulling a little chair about the garden. This recreation was attended with considerable exertion, and employed all her strength. A swelling, after a few weeks' residence, being discovered in the lower part of the back, though scarcely perceptible and unattended with pain, the mother hurried her away to London. The same surgeon being again consulted, he recommended exercise in the open air, nutritive diet, and tonic medicines. He said, the crookedness of the spine could not be removed. She was accordingly sent to the sea, and remained there till late in the autumn. The swelling and other symptoms continuing to increase during the winter, another hospital surgeon was applied to: he advised issues and rest for twelve months, promising a complete cure, if his directions were properly observed.

Issues were accordingly made on each side of the projecting bone in February last, and a discharge kept up by the usual means rather more than two months. They were discontinued early in May, because the swelling appeared to get larger during the discharge. She is exceedingly languid and fretful; is very restless, and complains of wandering pains in the belly. Urine pale and turbid; bowels costive; pulse quick and small; appetite very bad.—*July* 1*st*, 1819.

The skin over and near to the affected bones of the back were ordered to be well rubbed for half an hour daily. During the first friction, the upper and middle bones became visibly depressed; and the servant was sensible of considerable motion in the ribs and vertebræ, while rubbing them. This was clearly seen by myself and three other persons. A grating noise was also distinctly heard, which we imputed to the friction of the bones against each other. The servant then remarked, that she had frequently observed to her mistress that the bones varied in their height at different times.—*July* 2*d*.

The rounded form of the back is much reduced, and it is more shapely. There is already less hollowness, and the bone stands out only half an inch. All the other bones have apparently gone down.

The back has nearly recovered its natural shape and firmness. The noise and motion of the bones are no longer perceptible. The projection is visibly reduced.—*Sept.* 10*th.*

The form of her back has recovered its natural shape, which is particularly fine. Only the eleventh dorsal vertebra, the first that projected, is in the least displaced, and the rise is scarcely visible. She has grown full two inches in length since July, though she had increased nothing in the preceding two years. Pulse regular and stronger; bowels and urinary secretion natural.—*Oct. 7th,* 1819.

*Sixth Case.*

Frederick G. Pratt, of Camden-Town, aged two years and five months, was struck in the loins by the fore-wheel of a stage-coach, the 13th of last December. His lower limbs have ever since been insensible, and incapable of voluntary motion. Several months ago, a hot poker burnt his right leg in two places: he felt no pain at the time, nor the dressings afterwards; a thick escar was gradually separated, and the sores healed very slowly. His feces and urine have ever since the accident been discharged involuntarily, and only when he cries. The urine, for the first two or three days, required to be taken away by a catheter. His stools are extremely offensive, and have, as the parents call it, a rotten odour. The urine, especially what is received upon cloths during the night, his mother observes, smells like spirits of hartshorn, and suffuses her eyes with moisture when she removes the cloths. He was first observed to hesitate in speaking little more than two months since; and the impediment has so much increased, that he has lately begun to stammer. He is generally cheerful, and continues to grow, except in the lower extremities: these are wasted, are extremely flaccid, and quite loose. When put in action by external means, their motions resemble more those of an automaton in wires, than of limbs endowed with vitality.

On examination, I found the first lumbal vertebra wholly dislocated, and driven into the left loin. The right transverse process is sunk downwards; the opposite one has risen, and can be distinctly felt below the skin. The last dorsal and second lumbar vertebræ were also displaced by the accident. Several other dorsal bones are gradually suffering spontaneous luxation, from the want of vertebral support below them.—*Sept. 22d,* 1820.

## Figures and Tables

**Fig 1 Fig 2 f1:**